# Methicillin-sensitive *Staphylococcus aureus* bacteremia in aged patients: the importance of formal infectious specialist consultation

**DOI:** 10.1007/s41999-018-0038-2

**Published:** 2018-03-06

**Authors:** E. Forsblom, A. Kakriainen, E. Ruotsalainen, A. Järvinen

**Affiliations:** Division of Infectious Diseases, Inflammation Center, University of Helsinki and Helsinki University Central Hospital, Aurora Hospital, Nordenskiöldinkatu 26, Building 5, P.O. Box 348, 00029 HUS Helsinki, Finland

**Keywords:** *S. aureus* bacteremia, High age, Infectious specialist consultation, Deep infection

## Abstract

**Background:**

Infectious specialist consultations (ISC) provide ever more evidence for improved outcome in *Staphylococcus aureus* bacteremia (SAB). Most ISC are formal (bedside). However, the impact of ISC on clinical management and prognosis lacks evaluation in aged patients with SAB.

**Methods:**

Multicenter retrospective analysis of methicillin-sensitive (MS) SAB. Patients were stratified according to age ≥ 60 years (sub-analyses for ≥ 75 years and females) and formal (bedside) ISC given within 7 days of SAB diagnosis. The impact on management and outcome of formal ISC was explored. Statistics were performed with univariate analysis, Cox proportional hazards regression model analysis, including propensity-score adjustment, and graphic Kaplan–Meier interpretation.

**Results:**

Altogether 617 patients were identified and 520 (84%) had formal ISC. Presence of formal ISC resulted in equivalent clinical management regardless of age over or under 60 years: localization and eradication of infection foci (80 vs. 82% and 34 vs. 36%) and use of anti-staphylococcal antibiotics (65 vs. 61%). Patients aged ≥ 60 years managed without formal ISC, compared to those with formal ISC, had less infection foci diagnosed (53 vs. 80%, *p* < 0.001). Lack of formal ISC in patients aged ≥ 60 years resulted in no infection eradication and absence of first-line anti-staphylococcal antibiotics. Formal ISC, compared to absence of formal ISC, lowered mortality at 90 days in patients aged ≥ 60 years (24 vs. 47%, *p* = 0.004). In Cox proportional regression, before and after propensity-score adjustment, formal ISC was a strong positive prognostic parameter in patients aged ≥ 60 years (HR 0.45; *p* = 0.004 and HR 0.44; *p* = 0.021), in patients aged ≥ 75 years (HR 0.18; *p* = 0.001 and HR 0.11; *p* = 0.003) and in female patients aged ≥ 75 years (HR 0.13; *p* = 0.005).

**Conclusion:**

Formal ISC ensures proper active clinical management irrespective of age and improve prognosis in aged patients with MS-SAB.

## Introduction

*Staphylococcus aureu*s causes severe community- and healthcare-acquired bacteremia (SAB) [1, 2]. Despite anti-staphylococcal agents, new potent antimicrobials, and identification and eradication of infection foci [3, 4], the mortality in SAB remains high and ranges up to 30% [5]. Consultation by infectious specialist (ISC) provides evidence for improved clinical management of SAB. ISC enhances choice and duration of antibiotic treatment [6] and accelerate radiological diagnostics [7], localization [8] and eradication [9] of infection foci. Most ISC are given formal (bedside) and the superiority of formal ISC over other forms, e.g. informal (telephone) ISC, has been demonstrated [10]. However, most important, ISC improve outcome of SAB [7–10].

There is a rapid expansion of the geriatric population in the Western countries [11] with rising incidences of SAB among older persons [12, 13]. Specific characteristics have been identified: aged SAB patients are comorbid, bacteremia often hospital-acquired and the mortality high [14, 15]. However, few studies have compared clinical management and prognosis of aged versus young SAB patients and in many reports parameters that may influence prognosis, e.g. essential diagnostics, are unreported or performed rarely and deep infection foci documented scarcely or not identified [16–19]. The presence of ISC has been recorded in only three studies on aged patients with SAB reporting that patients were managed in conjunction with an ISC or that an ISC assisted in evaluating the source of SAB [14, 18], whereas one report concluded that aged patients were less likely to receive ISC guidance [19]. However, despite certification that ISC improve prognosis, the nature of ISC and the impact of ISC on clinical management and outcome have been neglected in previous reports on aged patients with SAB [14–19].

The objective was to explore how formal ISC impacts clinical management and prognosis of aged patients with methicillin-sensitive (MS) SAB. Main analyses were performed with patients aged ≥ 60 years and sub-analyses with patients aged ≥ 75 years and aged female patients. The inclusion of MS-SAB only enabled a setting where each patient received appropriate antimicrobial therapy and thus differences in empirical antibiotic therapy could be avoided.

## Materials and methods

### Study population

Multicenter and retrospective study with 90 days follow-up was performed. All adult patients with at least one blood culture for MS-SAB from five universities and seven central hospitals in Finland during January 1999–May 1999 and January 2000–August 2002 were recruited. Furthermore, as an extension, each adult patient with positive blood culture for *S. aureus* from Helsinki University Hospital in Finland in 2006–2007 was included. Two time-periods were mandatory to account for possible temporary differences in treatment management. Documentation included: gender, age, diseases, infection acquisition, illness severity, antibiotic therapy, infection foci, ISC, outcome and autopsy results. The primary outcome was mortality at 90 days. Secondary outcome measures were identification and eradication of infection foci and time to defervescence. Exclusions were: age < 18 years, pregnancy, epilepsy, bacteremia 28 days prior to the study and poly-microbial bacteremia.

### Definitions

Healthcare-acquired SAB was defined as bacteremia with the first positive blood culture for *S. aureus* received (1) ≥ 48 h after hospital admission or (2) within 48 h of hospital admission with a preceding previous hospital discharge within 1 week. Diseases were classified with McCabe’s criteria [20]. Severe sepsis was categorized as sepsis in combination with hypotension, hypo-perfusion or organ failure [21]. Formal ISC was defined as bedside consultation within 1 week of SAB diagnosis by infectious specialist including: review of patient records and physical investigation with comprehensive written directives into patient records on clinical management.

### Antibiotic therapy

The standard antibiotic therapy was anti-staphylococcal penicillin cloxacillin. Patients with penicillin contradictions received cephalosporin, either cefuroxime of ceftriaxone, as a first-line alternative therapy, or vancomycin, clindamycin or a carbapenem as second-line choice. Rifampicin and/or fluoroquinolone were provided as an adjunctive therapy. Antibiotic therapy was regarded correct when given intravenously for at least 28 days for a deep infection focus and 14 days in the absence of deep infection. Detailed information on indications, dosages and administration routes for antibiotic therapy has been provided in previous studies [22].

### Statistical analyses

Categorical variables were compared with Pearson’s *X*^2^ test and non-categorical variables with Student’s *t*-*test*. Odds ratios (OR) and hazard ratios (HR) with 95% confidence intervals (CI) were calculated. Univariate factors with *p* < 0.1 were entered into Cox regression model (proportional hazards regression). Furthermore, a propensity-score adjusted Cox regression model analysis was performed to evaluate the explicit prognostic impact of formal ISC in aged patients. First, propensity-scores were estimated by logistic regression and variables with significant connection to ISC identified. Second, a propensity-score adjusted Cox regression analysis was performed for prognostic parameters of 90-day outcome. Tests were two-tailed and *p* < 0.05 considered significant. Analyses were done with SPSS 12.0 (SPSS Inc., Chicago, IL, USA).

## Results

### Patient characteristics

**Fig. 1:**
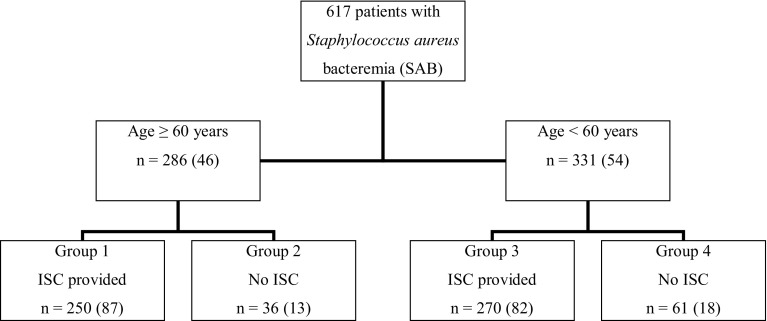
Study profile. Presentation of 617 methicillin-sensitive *Staphylococcus aureus* bacteremia patients stratified according to break-point age of 60 years and presence of formal (bedside) infectious specialist consultation (ISC) into different groups (groups 1–4)

A total of 617 SAB patients were identified. Altogether 46% (286) were aged ≥ 60 years. Formal ISC was provided to 87% (250) of patients aged ≥ 60 years and 82% (270) of patients aged < 60 years (Fig. 1). When comparing patients aged ≥ 60 years according to presence of formal ISC, no differences were seen regarding gender, SAB acquisition, previous hospitalization or underlying diseases. However, among patients receiving formal ISC, those aged ≥ 60 years, compared to those < 60 years, had more healthcare-acquired SAB (63 vs. 44%), more previous hospitalization (62 vs. 42%) and less healthy or non-fatal underlying diseases (61 vs. 85%) (*p* < 0.001). A total of 9% (56) had severe sepsis at blood culture collection. No differences in severe sepsis were observed for patients aged over or under 60 years when compared according to presence of formal ISC (Table 1).

**Table 1:** Patient demographics, severity of illness, clinical management and outcome in 617 methicillin-sensitive *Staphylococcus aureus* bacteremia patients stratified according to break-point age of 60 years and presence of formal (bedside) infectious specialist consultation (ISC)

Patient characteristics	Group 1 ≥ 60 years Formal ISC present *n* = 250	Group 2 ≥ 60 years Formal ISC absent *n* = 36	Group 3 < 60 years Formal ISC present *n* = 270	Group 1 vs. group 2		Group 1 vs. group 3	
				OR (95% CI)	*p* value	OR (95% CI)	*p* value
Demographics							
Male sex	144 (58)	23 (64)	182 (67)	0.77 (0.37–1.59)	0.47	0.65 (0.46–0.94)	0.21
Age, years^a^	72.6 ± 7.6	71.1 ± 7.9	43.0 ± 12.3	–	0.29	–	< 0.001
Healthcare-acquired	157 (63)	19 (53)	118 (44)	1.51 (0.75–3.05)	0.25	2.18 (1.53–3.09)	< 0.001
Previous hospitalization^b^	154 (62)	25 (69)	114 (42)	0.71 (0.33–1.50)	0.36	2.19 (1.55–3.12)	< 0.001
Healthy-nonfatal^c^	153 (61)	19 (53)	230 (85)	1.41 (0.69–2.85)	0.34	0.27 (0.18–0.42)	< 0.001
Severe sepsis^d^	21 (8)	5 (14)	18 (7)	0.57 (0.20–1.62)	0.28	1.28 (0.67–2.47)	0.45
Infection foci							
Deep infections	200 (80)	19 (53)	222 (82)	3.58 (1.74–7.38)	< 0.001	0.87 (0.56–1.34)	0.52
Pneumonia	99 (40)	12 (33)	103 (38)	1.31 (0.63–2.74)	0.47	1.06 (0.75–1.51)	0.73
Endocarditis	39 (16)	2 (6)	47 (17)	3.14 (0.73–13.6)	0.11	0.88 (0.55–1.39)	0.58
Osteomyelitis^e^	94 (38)	3 (8)	110 (41)	6.63 (1.98–22.2)	0.001	0.87 (0.61–1.24)	0.44
Deep abscesses	85 (34)	2 (6)	143 (53)	8.76 (2.05–37.3)	0.001	0.46 (0.32–0.65)	0.001
Any foreign body infection	59 (24)	4 (11)	27 (10)	2.47 (0.84–7.27)	0.09	2.78 (1.69–4.55)	0.001
Infected PVC or CVC^f^	45 (18)	0	32 (12)	–	–	1.48 (0.89–2.44)	0.12
Clinical management							
TTE echocardiography^g^	165 (66)	19 (53)	174 (64)	1.74 (0.86–3.51)	0.12	1.07 (0.75–1.54)	0.71
TEE echocardiography^g^	55 (22)	2 (6)	54 (21)	4.79 (1.12–20.6)	0.021	1.13 (0.74–1.72)	0.58
Any infection foci removal	84 (34)	0	94 (36)	–	–	0.95 (0.66–1.36)	0.77
Heart valve replacement	2 (< 1)	0	4 (1)	–	–	0.54 (0.09–2.95)	0.47
Infected joint lavage	7 (3)	0	6 (2)	–	–	1.27 (0.42–3.82)	0.67
Standard antibiotics							
Cloxacillin	163 (65)	0	165 (61)	–	–	1.19 (0.83–1.70)	0.33
Cephalosporin	70 (28)	27 (75)	85 (31)	0.13 (0.06–0.29)	< 0.001	0.85 (0.58–1.23)	0.39
Other antibiotics	17 (7)	9 (25)	20 (7)	4.57 (1.87–11.2)	< 0.001	1.09 (0.56–2.14)	0.79
Treatment duration^h^	24.6 ± 13	19.8 ± 15	26.1 ± 12	–	0.038	–	0.19
Adjunctive antibiotics							
Fluoroquinolone	129 (52)	11 (31)	136 (50)	2.42 (1.14–5.14)	0.018	1.05 (0.75–1.48)	0.78
Rifampicin	152 (61)	11 (31)	175 (65)	3.53 (1.66–7.49)	0.001	0.84 (0.59–1.20)	0.34
Outcome							
Hospital duration^h^	38.7 ± 34	31.6 ± 26	32.7 ± 29	–	0.09	–	0.10
Mortality in 28 days	46 (18)	11 (31)	12 (4)	0.51 (0.24–1.12)	0.088	4.85 (2.50–9.39)	< 0.001
Mortality in 90 days	61 (24)	17 (47)	21 (8)	0.36 (0.18–0.74)	0.004	3.83 (2.25–6.51)	< 0.001

Altogether 61 (18%) patients were aged < 60 years and received no formal ISC and are omitted in the presentation. Values are expressed as number of patients (%), odds ratios (ORs) with 95% confidence intervals (CI) and mean ± standard deviation (SD)

^a^Student’s *t* test (mean ± SD)

^b^Within 2 months preceding SAB

^c^McCabe’s classification [20]

^d^At blood culture collection

^e^Including septic arthritis

^f^Peripheral or central venous catheter

^g^Thoracic or –esophageal

^h^Days (mean ± SD)

### Deep infection foci

Deep infection foci were identified in 75% (463) of patients. In patients aged ≥ 60 years, presence of formal ISC, compared to lack of formal ISC, resulted in more deep infection foci (80 vs. 53%, *p* < 0.001), osteomyelitis or septic arthritis (38 vs. 8%) and deep abscesses (34 vs. 6%) (*p* = 0.001) localized. However, when comparing formal ISC for patients aged ≥ 60 years with those aged < 60 years, no difference was seen in the presence of deep infection foci (80 vs. 82%) or peripheral or central venous catheters (18 vs. 12%), whereas deep abscesses were encountered less (34 vs. 53%, *p* = 0.001) and foreign body infections more (24 vs. 10%, *p* = 0.001) (Table 1).

### Clinical management

Trans-thoracic and trans-esophageal echocardiography (TTE and TEE) were provided to 64% (183) and 20% (57) of patients aged ≥ 60 years. No difference in access to TTE was seen according to age over or under 60 years or presence or absence of formal ISC. However, in patients aged ≥ 60 years formal ISC, compared to lack of formal ISC, resulted in more TEE (22 vs. 6%, *p* = 0.021). Formal ISC resulted in active infection eradication in patients aged ≥ 60 years including infection removal (34%), heart valve replacement (< 1%) and joint lavage (3%). Formal ISC gave access to infection foci removal irrespective of age over or under 60 years. However, infection eradication was not provided to patients aged ≥ 60 years managed without formal ISC (Table 1).

### Antibiotic therapy

All patients were provided with an intravenous standard antimicrobial agent effective in vitro against *S. aureus* blood isolate from the first day of the positive blood culture: 53% (327) received standard antibiotic cloxacillin, 37% (228) cefuroxime or ceftriaxone and 2% (12) vancomycin. The remaining received clindamycin or a carbapenem. Adjunctive rifampicin and fluoroquinolone therapy was provided to altogether 57 and 49% of patients aged ≥ 60 years, respectively. The total intravenous antibiotic duration for patients aged ≥ 60 years receiving formal ISC was 24.6 ± 13 (days ± SD) and for patients managed without formal ISC 19.8 ± 15 (days ± SD) (*p* = 0.038). Formal ISC resulted in equal treatment with cloxacillin, cephalosporin and adjunctive antibiotics fluoroquinolone or rifampicin for patients aged over or under 60 years. None of patients aged ≥ 60 years that were managed without formal ISC received cloxacillin as first-line antibiotics. Moreover, administration of cephalosporins or other antibiotics as first-line choice was less common among patients aged ≥ 60 receiving formal ISC compared to those managed without formal ISC (28 vs. 75% and 7 vs. 25%, *p* < 0.001) (Table 1).

### Time to defervescence

**Fig. 2:**
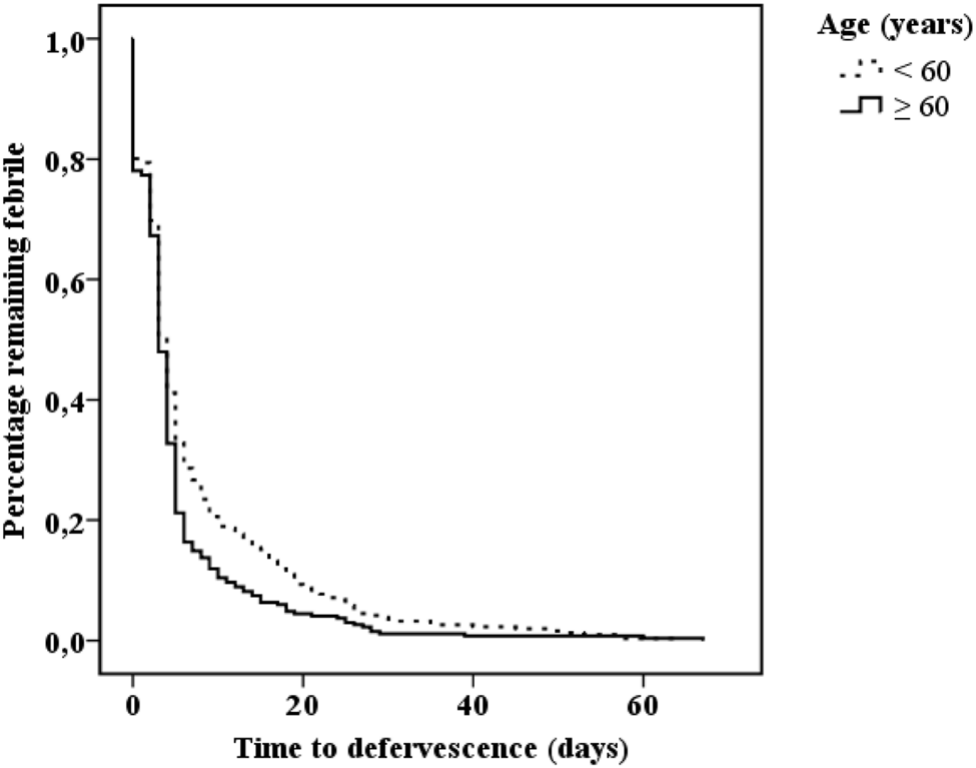
Kaplan–Meier analysis for time to defervescence (days) in 578 methicillin-sensitive *Staphylococcus aureus* bacteremia patients categorized according to the break-point age 60 years. Log rank = 0.006

Data for time to defervescence was retrieved for 578 patients. Patients aged ≥ 60 years, compared to patients aged < 60 years, had shorter mean time to defervescence (days ± SD) (5.1 ± 7.7 vs. 7.2 ± 10.1) (*p* = 0.006) (Fig. 2). The impact of clinical management on defervescence in aged patients was explored by categorizing time to defervescence according to cut-off value of 7 days. However, formal ISC and localization or eradication of infection did not affect fever time in patients aged ≥ 60 years (Table 2).

**Table 2:** The impact of clinical management on time to defervescence stratified according to cut-off value of 7 days in 268 methicillin-sensitive *Staphylococcus aureus* bacteremia patients aged ≥ 60 years

Clinical management	Time to defervescence			
	< 7 days *n* = 227 (85)	≥ 7 days *n* = 41 (15)	OR (95% CI)	*p* value
Formal ISC	209 (92)	35 (85)	1.99 (0.74–5.36)	0.17
Deep infection	173 (76)	35 (85)	0.55 (0.22–1.38)	0.19
Endocarditis	34 (15)	7 (17)	0.86 (0.35–2.09)	0.73
Pneumonia	86 (38)	19 (46)	0.71 (0.36–1.38)	0.31
Osteomyelitis^a^	81 (36)	14 (34)	1.07 (0.53–2.16)	0.85
Foreign body infection	52 (23)	8 (20)	1.23 (0.53–2.82)	0.63
Surgical-radiological infection removal	74 (33)	10 (24)	1.49 (0.69–3.22)	0.29
Heart valve replacement	2 (1)	0	–	–
Infected joint lavage	7 (3)	0	–	–

Values are expressed as number of patients (%) and odds ratios (ORs) with 95% confidence intervals (CI) or mean ± standard deviation according to Student’s *t* test

^a^Including septic arthritis

### Outcome

The total hospital duration for patients aged ≥ 60 years receiving formal ISC was 38.7 ± 34 (days ± SD) and for patients managed without formal ISC 31.6 ± 26 (days ± SD) (*p* value = 0.09). The overall case fatality in 617 patients was 13% at 28 days and 19% at 90 days. Patients aged ≥ 60 years receiving formal ISC, compared to those without, had lower mortality at 28 days (18 vs. 31%, *p* = 0.088) and at 90 days (24 vs. 47%, *p* = 0.004) (Table 1). Parameters for 90-days mortality in patients aged ≥ 60 years were evaluated (Table 3a, b). In univariate analysis, factors with positive prognosis were lack of underlying diseases (OR 0.27, *p* < 0.001), formal ISC (OR 0.36, *p* = 0.004) and adjunctive rifampicin therapy (OR 0.31, *p* < 0.001), whereas poor prognosis connected to severe sepsis (OR 2.52, *p* = 0.023), endocarditis (OR 2.41, *p* = 0.01) and pneumonia (OR 3.98, *p* < 0.001) (Table 3a). In Cox proportional regression analysis lack of underlying diseases (HR 0.38, *p* < 0.001), formal ISC (HR 0.45, *p* = 0.004) and adjunctive rifampicin therapy (HR 0.32, *p* < 0.001) were positive prognostic parameters, whereas severe sepsis (HR 1.98, *p* = 0.039), endocarditis (HR 2.09, *p* = 0.01) and pneumonia (HR 2.58, *p* < 0.001) were associated to poor prognosis (Table 3). When Cox proportional regression was repeated by including only patients aged ≥ 75 years or including only female patients aged ≥ 75 years, the positive prognostic impact of formal ISC remained (HR 0.18, *p* = 0.001 and HR 0.13, *p* = 0.005).

**Table 3:** Cox proportional regression for prognostic factors of 90-day mortality in methicillin-sensitive *Staphylococcus aureus* bacteremia patients aged ≥ 60 years (*n* = 286)

Patient characteristics	Outcome		Univariate analysis		Cox regression	
	Died *n* = 78 (27)	Survived *n* = 208 (73)	OR (95% CI)	*p* value	HR (95% CI)	*p* value
Male sex	46 (59)	121 (58)	1.03 (0.61–1.75)	NS	–	–
Healthcare-acquired	48 (62)	128 (62)	1.00 (0.59–1.71)	NS	–	–
Healthy—nonfatal^a^	29 (37)	143 (69)	0.27 (0.16–0.46)	< 0.001	0.38 (0.24–0.61)	< 0.001
Severe sepsis^b^	12 (15)	14 (7)	2.52 (1.11–5.72)	0.023	1.98 (1.04–3.79)	0.039
Formal ISC^c^	61 (78)	189 (91)	0.36 (0.18–0.74)	0.004	0.45 (0.26–0.78)	0.004
Endocarditis	18 (23)	23 (11)	2.41 (1.22–4.77)	0.01	2.09 (1.19–3.64)	0.01
Pneumonia	49 (63)	62 (30)	3.98 (2.30–6.88)	< 0.001	2.58 (1.59–4.18)	< 0.001
Rifampicin^d,e^	20 (26)	110 (53)	0.31 (0.17–0.55)	< 0.001	0.32 (0.19–0.54)	< 0.001
Fluoroquinolone^d^	35 (45)	105 (50)	0.79 (0.47–1.35)	NS	–	–

Values are expressed as number of patients (%) and odds ratios (OR), hazards ratio (HR) and 95% confidence intervals (95% CI) are presented

*NS* non-significant

^a^McCabe’s classification [20]

^b^At blood culture collection time-point

^c^Infectious specialist consultation

^d^Adjunctive therapy

^e^For at least 14 days

In propensity-score-adjusted Cox proportional analysis, parameters for 90-days outcome were: formal ISC (HR 0.44, *p* = 0.021), lack of underlying diseases (HR 0.37, *p* < 0.001), severe sepsis (HR 1.99, *p* = 0.039), endocarditis (HR 2.06, *p* = 0.014), pneumonia (HR 2.57, *p* < 0.001) and rifampicin adjunctive therapy (HR 0.31, *p* < 0.001) (Table 4). When propensity-score-adjusted Cox proportional regression was repeated by including only patients aged ≥ 75 years or including only female patients aged ≥ 75 years, the impact of formal ISC remained (HR 0.11, *p* = 0.003 and HR 0.13, *p* = 0.005).

**Table 4:** Propensity-score-adjusted Cox proportional regression analysis for 90-day mortality in methicillin-sensitive *Staphylococcus aureus* bacteremia patients aged ≥ 60 years (*n* = 286) according to infectious specialist consultation

Patient characteristics	Propensity-score-adjusted multivariate analysis HR (95% CI)	*p* value
Absence of formal ISC^a^	1.0	–
Presence of formal ISC^a^	0.44 (0.22–0.88)	0.021
Healthy—nonfatal^b^	0.37 (0.23–0.60)	< 0.001
Severe sepsis^c^	1.99 (1.03–3.81)	0.039
Endocarditis	2.06 (1.16–3.67)	0.014
Pneumonia	2.57 (1.58–4.17)	< 0.001
Rifampicin^d,e^	0.31 (0.18–0.54)	< 0.001

^a^Infectious specialist consultation

^b^McCabe’s classification [20]

^c^At blood culture collection time-point

^d^Adjunctive therapy

^e^For at least 14 days

To further evaluate differences between patients aged over or under 60 years, autopsy results were examined for altogether 57 patients. The immediate causes of death were stratified according to age. For patients aged < 60 years (*n* = 15), the three most common immediate causes of death were acute subarachnoid or gastrointestinal hemorrhages (43%), severe sepsis (20%) or pneumonia (20%). For patients aged ≥ 60 years (*n* = 42), the corresponding causes were SAB with or without a background condition (43%), acute cardio-pulmonary disease (21%) or deep infection (19%). Altogether, the immediate causes of death were infection related in 67% of patients aged ≥ 60 years and 60% in patients aged < 60 years.

## Discussion

The main observations were that formal ISC ensures proper clinical management irrespective of age and formal ISC improves prognosis of aged patients with MS-SAB. Accounting for all prognostic parameters, patients aged ≥ 60 years, patients aged ≥ 75 years and female patients aged ≥ 75 years, had a more than twofold lower odds ratio for a fatal outcome due to formal ISC.

Previous reports connect ISC to improved antibiotic therapies, accelerated diagnostics and eradications of deep infection foci and better outcomes in SAB [6–10, 23]. Corresponding observations were seen in the present study. We provided formal ISC within 7 days of SAB diagnosis and, among patients aged ≥ 60 years, this resulted in echocardiography in up to 66%, deep infection foci identification in 80% and infection eradication provided to 34% of patients. Formal ISC resulted in radiological infection diagnostics, localization and eradication irrespective of age over or under 60 years. However, patients aged ≥ 60 years managed without formal ISC had less infection foci diagnosed and were provided no infection foci eradications compared to those receiving formal ISC. Similar trends were observed also for antibiotics. Patients aged ≥ 60 years managed by formal ISC had anti-staphylococcal penicillin as first-line alternative in 2/3 of cases, whereas cephalosporins or other antibiotics were used in under 1/3 and adjunctive antibiotics in over 50% of cases. Most important, formal ISC resulted in antibiotic therapies irrespective of age over or under 60 years. Contrary, no of patients aged ≥ 60 years managed without formal ISC had anti-staphylococcal penicillin as first-line antibiotics, whereas 3/4 had cephalosporins as first-line alternative and adjunctive antibiotics were provided in only 1/3 of cases.

Deep infection foci have been identified in only 14–31% of aged patients in earlier studies on SAB [14–16, 18, 24]. Previous reports have stated that aged SAB patients are less likely to receive ISC, echocardiography or infection foci identification or eradication, and extensive diagnostics have not been pursued in aged patients due to presumed poor outcome [17, 19, 24, 25]. To the best of our knowledge, only three studies report presence of ISC stating that patients were managed in conjunction with an ISC [14, 18], whereas one report concluded that aged patients were less likely to receive ISC guidance [19]. However, the content or nature of ISC or the impact of ISC on disease progression or prognosis was not evaluated [14, 18, 19]. The importance of infection identification and eradication in SAB has been confirmed [4, 26] and lack of echocardiography and undiagnosed infection foci have been suspected to connect to mortality in aged SAB patients [14, 17]. Autopsy examinations in the present study revealed that for deceased patients both bacteremia and deep infection foci account for a larger part of mortalities of aged patients compared to young ones. The present study demonstrate that formal ISC-guided clinical management result in frequent localization and eradication of deep infection with subsequent positive prognostic impact in aged patients with SAB.

Comparison of prognosis in aged and young SAB patients is challenging due to differences in age categorizations and reported follow-up time and in earlier studies. We applied break-point ages of 60 and 75 years with mortality rates for patients aged over 60 years at 28 and 90 days of 18 and 24% for patients managed by formal ISC and 31 and 47% for patients not receiving formal ISC. Previous reports have presented results according to mean ages of 63–85 years [14, 15] or break-point age of 60–65 years [16, 18, 19]. In older patients, the mortalities have varied considerably with 15% at 7 days [15], 11–36% at 30 days [18, 24], 29% at 90 days [17] and 33–56% at 6 months [14]. The present study observed higher fatalities for aged patients compared to young. This is in line with earlier reports [16, 17, 19, 24] but deviate from one study with no link between age and clinical outcome [18]. Previous reports have presented age, comorbidity, MRSA and unknown bacteremia source as independent parameters for mortality among aged patients with SAB [14, 16, 17, 19, 24]. Parameters influencing outcome in patients aged ≥ 60 years in the present study have been identified earlier in SAB patient cohorts without age specification, i.e. comorbidity [5, 6], severity of illness [7], ISC [7–10], pneumonia or endocarditis [7, 10] and rifampicin therapy [22]. Moreover, adjunctive fluoroquinolone therapy did not impact outcome and this is in line with previous observations [22].

Previous studies on aged SAB patients do not report fever [14–16, 18, 24], whereas two studies reported that aged SAB patients were more likely to be afebrile prior to SAB [17, 19]. In the present study, defervescence was significantly shorter for aged patients compared to young. We have previously shown that ISC reduced fever duration in SAB [10]. However, in the present study clinical management or formal ISC had no impact on defervescence in patients aged ≥ 60 years. Hence, time to defervescence among patients aged ≥ 60 years does not seem to correlate with treatment strategies.

MRSA weakens prognosis [27] and MRSA in previous reports on aged SAB patients has been as much as 52–100% [14–16, 18]. We included only MS-SAB. Thus, patients had proper antibiotic therapy from the first day of positive blood culture excluding bias from differences in empirical antibiotic therapy. In the present study 1/2 of patients aged ≥ 60 years received adjunctive rifampicin therapy. We have shown that rifampicin adjunctive therapy may impact prognosis positively [22, 28]. However, among patients aged ≥ 60 years, the risk of drug interactions may have resulted in a selection bias favoring rifampicin treatment in less comorbid patients and hence, the potential positive prognostic impact of rifampicin in aged patients should be interpreted with caution. The authors recommend that prospective randomized clinical trials evaluate the potential advantage of rifampicin therapy before it can be routinely recommended as part of treatment of aged patients with SAB.

There are weaknesses in the present study that have to be accounted for when interpreting results. First, the retrospective design includes risk for bias due to differences in patient groups. However, propensity-score adjustment may reduce potential bias [29]. Second, aged infectious patients may lack fever and present non-specific symptoms. Moreover, declined functional status has been connected to mortality among aged infectious patients [30]. Corresponding data were not recorded. Third, the patient cohort of the present study was originally collected to investigate trends of nosocomial SAB and prognostic impact of fluoroquinolones, rifampicin and ISC [10, 12, 22, 28]. However, the authors noticed that, although the incidence of SAB among older persons is rising, the prognostic impact of ISC has not been extensively evaluated among aged patients with SAB. Regarding the earlier time-period of the patient cohort, the question of whether the patient data in the present study is valid to current clinical practice may be raised. Management of SAB is continuously developed, however, fundamental elements of SAB management remain unchanged over the years, e.g. the importance of proper non-delayed antibiotic therapy and diagnostics of infection foci. The authors view that the high presence of formal ISC has ensured recording of relevant data and high-standard management of SAB. Hence, the authors conclude that the patient data are not outdated for current clinical practice.

## Conclusion

Formal ISC ensures proper and active clinical management irrespective of age and improve prognosis in aged patients with MS-SAB. The authors encourage clinicians to manage aged patients with MS-SAB through formal ISC guidance.
