# Correction to: Methicillin-sensitive *Staphylococcus aureus* bacteremia in aged patients: the importance of formal infectious specialist consultation

**DOI:** 10.1007/s41999-018-0054-2

**Published:** 2018-04-13

**Authors:** E. Forsblom, A. Kakriainen, E. Ruotsalainen, A. Järvinen

**Affiliations:** Division of Infectious Diseases, Inflammation Center, University of Helsinki and Helsinki University Central Hospital, Aurora Hospital, Nordenskiöldinkatu 26, Building 5, P.O. Box 348, 00029 HUS Helsinki, Finland

## Correction to: European Geriatric Medicine 10.1007/s41999-018-0038-2

The article “Methicillin-sensitive *Staphylococcus aureus* bacteremia in aged patients: the importance of formal infectious specialist consultation”, written by E. Forsblom, A. Kakriainen, E. Ruotsalainen and A. Järvinen, was originally published electronically on the publisher’s internet portal (currently SpringerLink) on 06 March 2018 without open access.

With the author(s)’ decision to opt for Open Choice the copyright of the article changed on 10 April 2018 to © The Author(s) 2018 and the article is forthwith distributed under the terms of the Creative Commons Attribution 4.0 International License (http://creativecommons.org/licenses/by/4.0/), which permits use, duplication, adaptation, distribution and reproduction in any medium or format, as long as you give appropriate credit to the original author(s) and the source, provide a link to the Creative Commons license and indicate if changes were made. The original article has been corrected.

